# The Relationship of Diseases of the Bronchi and Lungs to Those of the Nose and Throat

**Published:** 1904-06

**Authors:** 


### THE RELATIONSHIP OF DISEASES OF THE BRONCHI AND LUNGS TO THOSE OF THE NOSE AND THROAT.*

In these days of specialism in medicine, the upper air-passages present a fertile field for observation and study. But no specialty can be divorced from the parent stock of medicine in general, and become, as it were, an appendage. It must take an active part in

*Read by Frank W. Thomas, Ph. M., M.D., Pomona, Cal., at the Southern California Medical Society, Redlands, December 2, 1903.

the solution of problems pertaining to the whole system as well. While on the other hand, the general practitioner should not fail to recognize symptoms of disease in special organs which might be of the greatest importance to him in the management of his cases.

The inborn sympathy that exists between the different parts of the human organism in the case of disease can not be ignored.

The relationship existing between many of the general diseases and those of the nose and throat is both interesting and important, either from a diagnostic or pathologic standpoint.

The upper air-passages, comprising the nose, pharynx and larynx, present a canal of varying form and diameter, lined in its entire extent, except when the respiratory and alimentary tracts cross each other in the pharynx, by mucous membrane, covered with ciliated columnar epithelium; so that nose, pharynx and larynx imperceptibly merge one into the other without the interposition of a sharp line of demarcation. It follows that pathological changes in any portions of the upper air-passages are not sharply limited in their local effects and ultimate consequences, but invade adjacent areas quite irrespective of the anatomic boundaries. It is well known that catarrhal affections, for instance, of the upper air-passages are not limited to a circumscribed area; they display, on the contrary, a peculiar descending character, as it is called, beginning in the nose as an acute rhinitis and invading at certain definite intervals the pharynx, larynx and bronchial tubes. With some of the infectious diseases, especially diphtheria, the case is quite different; the local manifestations may appear, first, either in the pharynx or in the nose, usually in the pharynx, as a matter of fact, and spread from that region either upward into the nose or downward into the larynx. Looking further down toward the lungs, we find a large influence exerted upon disease in those organs by irregularities of the upper air-passages.

A glance at the significance of the act of respiration is important in this connection. The air-passages should not be looked upon merely as canals for the transmission of inspired air, for each segment has a special function of its own, and contributes to the preparation of the air for reception into the lungs, and this function can not remain in abeyance without detriment to the organism.

For instance, the nose, the portal through which the air gains admittance to the body, is charged with the duty of preparing the air for entrance into the deeper air-passages, by removing foreign substances, by warming the air, and imparting to it the requisite degree of moisture, as well as to protect the organism by means of the sense of smell and the nasal reflexes. One of the most important of these functions is that of supplying the necessary moisture to the inspired air, a function which the mouth is unable to perform. The nose thus relieves the bronchi and lungs of an onerous duty, which falls on them to a much greater degree if respiration is performed through the mouth. To enable it to supply the required amount of moisture, the nose is endowed with unusual secretory activity, which, according to physiologists, is derived largely from the abundant supply of serous and mucous glands, and from an extensive system of lymphatics.

Mouth-breathing is generally regarded as injurious to good health. The oral cavity is not adapted to replacing the nose in the act of breathing. The width of the oral cavity is such that the air-current encounters no resistance, and conseqently its progress is not retarded as it is in the narrow passage of the nasal cavity, and no time is afforded for warmth, purification and saturation. The less abundant vascular supply, and the absence of cavernous tissue which regulates the temperature in the nose, and the absence of an abundant watery secretion in the oral cavity, show a different function. Besides, the epithelium in the mouth is of the squamous variety, and therefore incapable, in contradistinction to the ciliated columnar epithelium in the nose, of removing anatomically and physiologically any deleterious substance in the air-current. All these differences combine to make the mouth unfit to supply an air-current which would be other than injurious to the organism. Mouth-breathing naturally becomes a habit if obstruction is situated either in the nose or in the postnasal space. These obstructions may be from hyperplasias and tumors in the nose, structural anomalies, deviated septum, cystic formation, etc., which occlusion of the posterior nares may be caused by tumors in the postnasal space, and especially by adenoid growths in the vault of the pharynx.

The evil effects of mouth-breathing first manifest themselves in the mucous lining of the pharynx and larynx, which become dry because the air has not been properly prepared and saturated. Dust particles are deposited first in the mucous membrane of the mouth and oral pharynx, and later make their way into the larynx and deeper air-passages. The constant irritation of the dry and impurified air coming in contact with the mucous membrane of the upper air-passages gives rise, as can readily be understood, to chronic catarrhal conditions. Thus it is found that mouth-breathers, as represented typically by children in the early stages of enlarged tonsils, are prone to become the subjects of catarrh of the upper air-passages, of recurring pharyngeal and laryngeal catarrh, and of acute bronchial catarrh; which, if the condition continues, usually develops chronic bronchitis that can seldom be permanently cured without restoring proper nasal respiration. This is particularly true in children of the so-called scrofulous habit, in whom the hypertrophy of the lymphatic elements in the postnasal space is followed by occlusion of the posterior nares.

In atrophic conditions of the nose, coupled, as they are, with metaplasia of the epithelium, foreign bodies and disease germs contained in the inspired air cling to the walls of the cavities, and eventually penetrate into the deep air-passages. This condition is particularly noticeable in persons whose occupation obliges them to breathe impure air laden with dust particles, whether mineral or vegetable, as, for instance, coal dust or stone dust, which is easily carried down with the inspiratory blast and lodged in the bronchi. In this way the various forms of pneumoconiosis, anthracosis and chalicosis are developed. The question naturally arises, if inspired air laden with dust and disease germs produces disease in certain instances, why does not every individual fall a victim to disease in whose nostrils and throat bacteria are found? Considering the number of bacteria contained in the air, and the great quantity of air that passes through the nose, we would naturally expect to find a very large number of micro-organisms in the nasal chambers, and, as a matter of fact, we do. Opinions are divided on the fate of the germs introduced into the nose, both as to the depth to which they penetrate into the nose and as to their behavior therein. Some investigators hold that the nose is a playground for all kinds of bacteria. Others have recently advanced the theory that the germs are arrested in the vestibule, and only in exceptional cases and in small numbers penetrate into the deeper portions of the nose. The question also naturally arises whether the nasal mucus has a bactericidal power or not. Some authorities believe that it has. At any rate, it must arrest the growth of the germs to a certain extent, and the soil is beyond doubt an unfavorable one for the development of micro-organisms in the majority of persons. The conditions in this respect are analogous to those found in the oral cavity, which contains even a greater abundance of bacteria. The mere presence of germs is evidently not in itself injurious to the nose. Other factors must be taken into account—the number and virulence of the pathogenic germs which gain entrance; the disposition of the individual and his ability to resist disease; the presence of other bacteria which either assist or retard the growth of pathogenic varieties.

Diseases of the lungs may owe their origin to direct extension of disease of the upper air-passages, as, for instance, chronic bronchitis may result from chronic atrophic catarrh, or from suppurative processes in the nose or its tributary cavities, or from the post-nasal space. Under such circumstances the bronchitis may prove very obstinate, especially if pus trickles down from the nasal pharynx into the deeper air-passages and sets up a chronic irritation which may extend to the trachea, bronchi, lungs or pleura. In cases of marked atrophy of the mucous membrane with ozena of the nose and pharynx, experience teaches us to expect not only chronic bronchitis, but also emphysema and asthmatic attacks. Such conditions are more frequently seen in elderly people who have for many years suffered from bronchitis due to ozena.

We pass next to note some of the changed conditions in the upper air-passages due to disease of the lungs. The most important alterations occur as a result of the irritation of the mucous membranes by the passage of the secretions. Any chronic disease of the lungs in which sputum is secreted is followed sooner or later by chronic laryngeal and pharyngeal catarrh, the intensity of which is in direct proportion to the amount and consistency of the expectorated material and to the amount of effort required to effect its expulsion. Hence, asthmatic and emphysematous patients, whose bronchi are filled with tenacious sputum which requires severe coughing and straining to remove, suffer more from inflammatory conditions of the upper air-passages than do those who have a simple bronchitis with watery secretions which they can expel without straining the muscles of the throat and neck.

A form of ascending catarrh of the air-passages has been described, beginning with bronchitis and terminating in acute laryngitis and pharyngitis. Emphysematous individuals suffer from congestive catarrh, and are prone to have hemorrhages.

Another interesting complication of lung diseases occasionally seen consists in paralysis of the larynx, the occurrence of which after disease of the lungs and pleura is explained by the course of the recurrent laryngeal nerve. The complications which form in the pleura over the apices in chronic inflammations, according to this theory, are very apt to include the nerve, especially on the right side, when such a complication is favored by the relation of the nerve to the subclavian artery; and, on the other hand, indurations of the apex may, during cicatrization, exert traction on the nerve. Such complications are found in chronic indurations of pleuritis or tuberculous consolidations.

Tubercular conditions found in the upper air-passages in connection with tuberculosis of the lungs presents an important complication, and the mode of infection in such cases has given rise to much discussion. Various opinions have been expressed with regard to the path by which the tubercle bacilli effect an entrance into the tissues. The mode of origin depends largely on whether the tuberculosis is considered as a primary or a secondary disease, since, if the pathogenic germs first become localized in the upper air-passages, the infection may be derived from the inspired air and the food digested; which, if we assume a primary tubercular focus in other organs, as, for instance, the lungs, secondary infection of the upper air-passages may take place either from within, by way of the lymphatic and vascular channels, or from without, by direct infection of the mucosa through the agency of tubercular sputa.

Formerly, primary tuberculosis of the upper air-passages was not believed to occur, but more recent investigations show that such may be the case, exceptional though it may be. Strassmann, German investigator, found tonsillar tuberculosis in thirteen out of twenty-one tubercular cadavers; another found twenty-one out of twenty-five cadavers. Others have given similar reports. Primary tuberculosis of the larynx must be regarded as rare, but post-mortem evidence has been produced to establish the possibility of its existence. The most frequent, not to say regular, form of infection met with in the upper air-passages must be the secondary one, brought about by direct contact with the infected sputum, or through the lymphatics and blood-vessels. In view of clinical experience and anatomical knowledge, an eminent authority, after discussing this subject, concludes that it seems more reasonable to recognize infection of the larynx by way of the lymph-channels, as probably most frequent; and infection by contact of sputum in those cases where there is erosion or ulceration of the mucous membrane of the larynx, pharynx, or nasal passages, as the case may be.—*Southern California Practitioner.*

